# Fibroblasts: the missing link between fibrotic lung diseases of different etiologies?

**DOI:** 10.1186/1465-9921-14-81

**Published:** 2013-08-02

**Authors:** Bruno Crestani, Valerie Besnard, Laurent Plantier, Keren Borensztajn, Arnaud Mailleux

**Affiliations:** 1 Inserm, U700, Paris 75018, France; 2 Univ. Paris Diderot, PRES Sorbonne Paris Cité, UFR de medicine, site Bichat, Paris, France; 3 APHP, Hôpital Bichat, Service de pneumologie A, Paris, France; 4 Département Hospitalo-universtaire FIRE (Fibrosis, Inflammation and Remodeling) and LabEx Inflamex, Paris, France; 5 APHP, Hôpital Bichat, Service d‘Explorations Fonctionnelles, Paris, France; 6 Service de Pneumologie A, Centre de compétence des maladies pulmonaires rares, Hôpital Bichat, 46 rue Henri Huchard, 75018, Paris, France

## 

Fibrotic lung disorders, either idiopathic, or associated with a specific etiology or a specific condition such as scleroderma, are increasingly recognized. As a whole they constitute a group of diseases characterized by the progressive destruction of the lung which ultimately leads to chronic respiratory failure and death. Improving the prognosis of these disorders requires the identification of drugs capable of inhibiting partially or totally the progression of lung fibrosis, and perhaps of reversing established fibrosis. This has been the focus of huge efforts from academic groups and pharma companies, and more than 20 different molecules are being investigated in clinical trials in idiopathic pulmonary fibrosis (IPF) a well defined and relatively frequent fibrotic lung disease of unknown etiology. However, until now, only one drug has been approved for lung fibrosis treatment. This drug, pirfenidone, has been shown to slow the decline of lung function in IPF, but no drug has demonstrated effects on survival in patients with lung fibrosis [1]. The effort must be prolonged and intensified.

Beside IPF, scleroderma-associated lung fibrosis is a well recognized fibrotic disorder. With pulmonary hypertension, lung fibrosis is now the main cause of death of patients with scleroderma [2]. The nature and pathophysiology of lung fibrosis in IPF and scleroderma are different. For instance, scleroderma affects mainly women, whereas IPF predominates in men; usual interstitial pneumonia is the pathological pattern of IPF, whereas non specific interstitial pneumonia is the main pattern in patients with scleroderma; the MUC5B promotor polymorphism is associated with IPF whereas it is not observed in patients with IPF [3]; IPF is rapidly progressive disorder as compared with the slowly moving scleroderma-associated lung fibrosis [2].

Fibrotic lung diseases are characterized by the pathological accumulation of fibroblasts, which are thought to be the main source of the extracellular matrix proteins which are accumulating in the fibrotic areas. The origin of fibroblasts is a matter of discussion, and the respective role of *1)* circulating precursors, of *2)* epithelial, mesothelial or endothelial to mesenchymal transition, and the role of *3)* local mesenchymal precursors has been suggested [4,5]. However, it has been shown that fibroblasts isolated from fibrotic lung have abnormal properties as compared with fibroblasts isolated from normal lung. For instance, fibroblasts have an increased capacity to produce extracellular matrix proteins such as collagen or fibronectin, a relative resistance to apoptosis, an increased capacity to secrete reactive oxygen species, and a reduced capacity to secrete anti-fibrotic molecules such as prostaglandin E2, fibroblast growth factor 7 or hepatocyte growth factor [5-7]. These profibrotic properties are maintained in vitro and have been linked at least in part to epigenetic changes such as increased DNA methylation or deacetylation, or abnormal micro-RNA network [8,9]. Targeting lung fibroblasts to treat lung fibrotic disorders might be a clue to the future. Therefore, evaluating lung fibroblasts to identify new fibrotic pathways carries important perspectives. In that way, Gisela Lindhal and colleagues recently compared the mRNA microarray profile of lung fibroblasts isolated from patients with IPF, systemic sclerosis, or controls [10]. They discovered the suppression of a group of interferon-stimulated genes, which was observed both in scleroderma fibroblasts and IPF fibroblasts. This observation, if confirmed by other groups, is very interesting, as it identifies a pathway that could be pharmacologically modulated. Many obstacles however do exist before this can reach the clinic. First, on the basis of blood and skin studies, scleroderma is currently considered to be an interferon-driven disease [11]. Why this could be different in lung fibroblasts from scleroderma-associated interstitial lung disease is difficult to understand now and will require specific studies. Second, sub-cutaneous interferon-gamma has been evaluated in the past in a randomized controlled multicentre trial in patients with scleroderma [12]. Tolerance was acceptable and there was a trend toward an improvement of skin sclerosis scores [12]. We also know from recent trials that systemic administration of interferon-beta [13] or interferon-gamma [14] does not influence the decline of lung function in patients with IPF. However some groups have suggested giving interferon through inhalation in order to increase the concentration in the lung and to somewhat reduce the systemic availability of the molecule [15]. Ten patients with IPF were prospectively treated with inhaled interferon-gamma for 80 weeks. The drug appeared to be safe [15]. Whether inhaled interferon-gamma could be useful in the long term in patients with IPF or scleroderma-associated interstitial lung disease deserves a rigorously evaluation in specific trials.

The results of Lindahl and colleagues point to an unexpected similarity of the microarray of IPF and scleroderma lung fibroblasts [10]. Although interesting, these results need to be confirmed as the number of IPF samples studied was small. However, such a similarity suggest that a drug targeting lung fibroblasts might work both in IPF and in scleroderma-associated lung fibrosis, and perhaps also in other fibrotic disorders.

Further studies are clearly needed, but this study illustrates the potential of modern screening methods to identify unexpected pathways in diseases [16].
